# Phenotypic Characterization of Non-toxigenic *Clostridioides difficile* Strains Isolated From Patients in Mexico

**DOI:** 10.3389/fmicb.2019.00084

**Published:** 2019-02-01

**Authors:** Margarita Camorlinga, Michelle Sanchez-Rojas, Javier Torres, Mariana Romo-Castillo

**Affiliations:** ^1^Unidad de Investigación Médica en Enfermedades Infecciosas y Parasitarias, Hospital de Pediatría, Centro Médico Nacional Siglo XXI, Instituto Mexicano del Seguro Social, Mexico City, Mexico; ^2^Facultad de Ciencia y Tecnología, Universidad Simón Bolívar, Mexico City, Mexico; ^3^CONACYT-IMSS, Unidad de Investigación Médica en Enfermedades Infecciosas y Parasitarias, Hospital de Pediatría, Centro Médico Nacional Siglo XXI, IMSS, Mexico City, Mexico

**Keywords:** phenotype, *Clostridioides difficile*, non-toxigenic, hospital-acquired diarrhea, Mexico

## Abstract

*Clostridioides difficile* is a Gram positive, sporulated, rod-shape, anaerobic pathogen responsible for nosocomial diarrhea and colitis, mainly in antibiotic treated patients. *C. difficile* produce two toxins responsible for disease, toxin A (TcdA) and toxin B (TcdB), although not all strains produce them. Non-toxigenic *C. difficile* (NTCD) strains are able to colonize the intestinal mucosa and are often isolated from asymptomatic individuals. NTCD are poorly studied, their evolutionary history has not been elucidated, and their relationship with illness remains controversial. The aim of this work was to analyze the phenotype of NTCD strains isolated from clinical cases in hospitals of México, and whether NTCD strains present characteristics that differentiate them from the toxigenic strains. Seventy-four *C. difficile* strains isolated from patients were tested for cytotoxicity and 14 were identified as NTCD strains. We analyzed phenotypical characteristics that are important for the biology of *C. difficile* like colony morphology, antibiotic resistance, motility, sporulation, and adherence. Strains were also genotyped to determine the presence of genes coding for TcdA, TcdB and binary toxin and ribotyped for 027 type. When compared with toxigenic strains, NTCD strains presented an enlarged branched colony morphology, higher resistance to metronidazole, and increased sporulation efficiency. This phenotype has been reported associated with mutations that regulates phenotypic characteristics like swimming, sporulation or adhesion. Our results show that phenotype of NTCD strains is heterogeneous but still present characteristics that differentiate them from toxigenic strains.

## Introduction

*Clostridioides difficile* has emerged as a healthcare problem worldwide, *C. difficile* colitis has been one of the most costly and common causes of diarrhea during the last 20 years, causing millions of deaths every year (Garey et al., 2010). The infection may present different clinical characteristics, including diarrhea, pseudomembranous colitis, fulminant colitis, toxic megacolon, and even death (Burke and Lamont, 2014). An important factor to consider in the transmission of *C. difficile* infection (CDI) is the asymptomatic carriage of strains, which has been reported in up to 18% of asymptomatic people (Donskey et al., 2015). To understand the mechanism by which this pathogen causes disease, it is necessary to know the ecology of the microorganism. *C. difficile* was considered as part of the normal intestinal microbiota, until 1970 when it was identified as an opportunistic pathogen. The administration of antibiotics disrupts the composition of gut microbiota causing a dysbiosis which results in reduced resistance to pathogens and C. *difficile* spores takes advantage of this to germinate and colonize the host.

*Clostridioides difficile* has several virulence factors, including toxins A and B encoded on a pathogenic island called PaLoc (Pathogenesis Locus) (Dingle et al., 2014). A third toxin, denominated CDT, which belongs to a family of toxins called binary toxins was recently identify in toxigenic *C. difficile* strains (Perelle et al., 1997; Gulke et al., 2001). The disruption of cytoskeleton, and cellular damage caused by the action of toxins A and B leads to the disassociation of thigh junctions between colonocytes, promoting the loss of epithelial integrity and consequently diarrhea (Hunt and Ballard, 2013). However, there are some strains that are unable to produce one or the two toxins and are denominated as Non-toxigenic *C. difficile* (NTCD). These NTCD are classified into three groups: the first does not contain the PaLoc genes, the second present a modified PaLoc and are unable to produce toxin A or B; finally, the third group produces very low amount of toxins and their cytotoxic activity is not detected (Fluit et al., 1991; Wren et al., 1993; Cohen et al., 1998; Brouwer et al., 2012; Natarajan et al., 2013). NTCD are usually isolated from asymptomatic individuals, but there are some reports on the association of NTCD strains with diarrhea (Martirosian et al., 2004). In addition, NTCD strains have been isolated from patients also infected with toxigenic strains (Miyajima et al., 2011; Behroozian et al., 2013) suggesting that NTCD may be involved in mixed infections. Other reports highlight the ability of NTCD to protect against toxigenic *C. difficile* strains, although the mechanisms providing this protective effect are unknown (Nagaro et al., 2013). Of note, recent works suggest that the use of NTCD strains as an oral probiotic would be an effective treatment for *C. difficile* associated diarrhea, an option probably simpler than fecal transplant (Villano et al., 2012; Nagaro et al., 2013; Gerding et al., 2015; Arruda et al., 2016). Thus, the role of NTCD strains in gut disease remains unclear and should be better studied. The aim of this study was to gain more information about phenotypic and genotypic characteristic of NTCD strains isolated from patients with hospital-acquired diarrhea.

## Materials and Methods

### Isolation and Identification of *C. difficile* From Patients

All samples were obtained from patients suffering from hospital-acquired diarrhea, attended at the National Medical Center “XXI Century” of the Mexican Institute of Social Security, Mexico City. Stools were cultured on CCFA agar after an ethanol shock and incubated under anaerobic conditions (N_2_, 5% CO_2_, and 5% H_2_) for 48–72 h at 37°C. Isolates were purified by passages of single colonies on Casman Blood Agar (Difco; Becton, Dickinson and Company, United States) and CCFA agar at least three times. *C. difficile* strains were identified by Gram staining, morphological growth in blood agar, UV-fluorescence and PCR amplification of *16S rRNA* and *tpi* genes. Isolates were also subcultured in meat broth (Difco; Becton, Dickinson and Company, United States) for storage.

The amplification of 16S rRNA gene was performed using primers PS13 and PS14 (Table 1), while *tpi* gene amplification was performed with *tpi*-Fw and *tpi*-Rv primers in a 25 μl reaction according to the GoTaq Green Master Mix Protocol (Promega, United States). Thermocycler conditions were 7 min at 95°C, followed by 30 cycles of 30 s at 94°C, 90 s at 57°C and 1 min at 72°C; then a final extension of 7 min at 72°C. Products were analyzed by 2.0% agarose electrophoresis stained with Eva Green 1X (Jena Bioscience, Germany).

**Table 1:** Oligonucleotides used in this study.

Name	Sequence (5′–3′)	Reference
*cdtB*-F1	TGG ACA GGA AGA ATA ATT CCT TC	Mulvey et al., 2010
*cdtB*-F2	TGC AAC TAA CGG ATC TCT TGC	Mulvey et al., 2010
*tcdA*-F	AGA TTC CTA TAT TTA CAT GAC AAT AT	Lemee et al., 2004
*tcdA*-RA3B	AAC ATC AAT CTCGAA AAG TCC AC	Lemee et al., 2004
*tcdB*-F3	AAT GCA TTT TTG ATA AAC ACA TTG	Murray et al., 2009
*tcdB*-F4	AAG TTT CTA ACA TCA TTT CCA	Murray et al., 2009
*tcdCEcoFw*	TAA ATA TCT AAT AAA AGG GAG AAT TCA TTA TGT TCT CTA AAA AAA ATG ATG GTA ACG	This work
*tcdCBamRv*	GCT GTA GAG AAA ATT AAT TAC TAT GGA TCC GTA TTA TAG TTA ATA TTT TAT ATT ATA GTC	This work
PS13	GGA GGC AGC AGT GGG GAA TA	Persson et al., 2008
PS14	TGA CGG GCG GTG TGT ACA AG	Persson et al., 2008
*tpi*-Fw	AAA GAA GCT AGT AAG GGT ACA AA	Lemee et al., 2004
*tpi*-Rv	CAT AAT TAA GGG TCT ATT CCT AC	Lemee et al., 2004

### DNA Extraction

Bacteria were cultured in BHI broth (Bacto; Becton, Dickinson and Company, United States) under anaerobically atmosphere for 24 h at 37°C. Then cultures were centrifuged and pellets were used for extraction of bacterial DNA using InstaGene Matrix (BioRad, Mexico) following manufacturer’s recommendations. DNA concentration and quality were measured using a Nanodrop spectrophotometer, ensuring that A260/280 values were greater than 1.8.

### Detection of Virulence Genes by PCR and Ribotyping

PCR amplification of the *tcdA*, *tcdB*, and *cdtB* genes was performed in 25 μl reaction with GoTaq Green Master Mix (Promega, United States), using the primers described in Table 1. Thermocycler conditions were 15 min at 95°C, followed by 30 cycles of 30 s at 94°C, 90 s at 57°C and 60 s at 72°C, with a final extension of 7 min at 72°C. We also designed primers to amplify the complete *tcdC* gene (Table 1) using the following conditions, 5 min at 94°C, followed by 30 cycles of 1 min at 94°C, 1 min at 42.5°C and 1 min at 72°C, with a final extension of 4 min at 72°C. Products were analyzed by 2.0% agarose electrophoresis, stained with Eva Green 1X (Jena Bioscience, Germany). Additional genotyping of the strains was done by ribotyping as described previously (Bidet et al., 1999). DNA from a clinical *C. difficile* strain ribotype 027 (Kindly donated by Dra. Elvira Garza, Monterrey, Mexico) (Camacho-Ortiz et al., 2015) was included in each run as a reference. Amplification products were subjected to electrophoresis in a 2.5% agarose gel, ethidium bromide stained and analyzed under ultraviolet light using the LabWorks Image Acquisition and Analysis Software (Version 4.5.00.0 for Windows, UVP).

### *In vitro* Cytotoxicity Assays

VERO (ATCC CCL-81) and HT-29 (ATCC HTB-38) cells were grown at a confluence of 70% in DMEM medium (In Vitro, Mexico) supplemented with 5% fetal calf serum (GIBCO, United States). The sensitivity of each cell line to the *C. difficile* toxins is different, VERO cells are more susceptible for toxin B, whereas HT-29 cells are more susceptible to toxin A (Torres et al., 1992). Briefly, *C. difficile* strains were grown 48 h in BHI media under anaerobic conditions. Then 50 μl of filtered supernatants were added onto monolayer of cells grown in 96-well plates under a 5% CO_2_ atmosphere. Cytotoxicity was recorded under the microscope after 24 and 48 h of incubation at 37°C. To confirm specificity of the cytotoxic effect, a neutralization assay with anti-toxin was done, the cytotoxic supernatants were incubated 1 h with the anti-toxin antibodies (Remel, United States) in a 1:1 ratio, before added to Vero cells. Neutralization of cytotoxicity was recorded after 24 h of incubation at 37°C under a 5% atmosphere.

### Colony Morphology

This assay was made as previously reported (Mackin et al., 2013) with slight modifications. Briefly, strains were grown overnight in *Brucella* broth at 37°C under anaerobic atmosphere. Then, 3 μl of culture were spotted onto BHI agar plates (Bacto; Becton, Dickinson and Company, United States) and incubated for 7 days to allow growth. Morphology of the single colonies was analyzed macroscopically. We used seven clinical toxigenic strains as controls (MX080, MX081, MX091, MX111, MX164, MX295, and MX300) as well as the ATCC 9689 and the 027/NAP1 *C. difficile* reference strains.

### Antimicrobial Susceptibility Test

Susceptibility of *C. difficile* strains to vancomycin, metronidazole, levofloxacin and clindamycin was evaluated using the E-test strip (Liofilchem, Italy) on Müller-Hinton Blood agar plates. Bacteria were grown on Brucella broth (BBL, Becton, Dickinson and Company, United States) for 48 h at 37°C, then bacterial concentration was adjusted to 0.5 McFarland turbidity and 100 μl of each inoculum were spread over the blood agar plate, and the corresponding E-test strip was place on the plate. Plates were incubated under an anaerobic atmosphere as previously described (Leroi et al., 2002). *C. difficile* ATCC 9689 and 027 strains were used as controls. Results were interpreted after 48 h of incubation in anaerobic conditions at 37°C. MIC interpretation was based on the values suggested by the European Committee on Antimicrobial Susceptibility (EUCAST) and by The Clinical and Laboratory Standards Institute (CLSI).

### Motility Assays

Swarming motility was analyzed as previously reported (Baban et al., 2013) with slight modifications. Briefly, *C. difficile* strains were grown for 18 h in Brucella broth under anaerobic conditions at 37°C. Bacterial concentration was adjusted to 0.5 McFarland turbidity and 3 μl were inoculated in previously reduced soft BHI agar (0.15% agar). Swarming motility was determined after 18 h of incubation under anaerobic conditions at 37°C. Assays were performed in triplicate for each strain in independent experiments.

### Sporulation Assays

A conventional spore recovery assay was used to evaluate the sporulation rate of the strains as previously reported, with some modifications (Garneau et al., 2014). C. *difficile* strains were grown on Brucella broth for 48 h anaerobically at 37°C, bacterial concentration was adjusted to 0.5 McFarland, and subject to alcohol shock by mixing 1.0 ml of bacterial suspension with 80% ethanol and incubated for 45 min. Samples were then diluted in Brucella broth and 10-fold serial dilutions were plated onto Cassman Blood Agar. Plates were incubated for 48 h anaerobically and then CFU were measured. Sporulation efficiency was determined using the following formula:

$$\mathrm{Sporulation efficiency}(\%)=\frac{\mathrm{Spores}}{\mathrm{Vegetative cells}+\mathrm{spores}}\times 100$$

### Adherence Assay

The ability of adherence of *C. difficile* strains were assayed on Vero cells. Vero cells were cultivated in culture bottles containing M199 medium (GIBCO, United States) supplemented with 5% fetal calf serum (GIBCO, United States), and grown until a confluence of 70%. Then cells were passed to a 96-well plates and cultured until a confluence of 90%. The cells were washed three times with PBS (Merk, Germany), and infected with *C. difficile* strains (10^7^ bacteria/mL). Infected cells were incubated for 1 h under anaerobic conditions at 37°C, and washed four times with PBS to removed non-adherent bacteria. Attached bacteria were then removed with 100 μL of 0.06% Triton X-100 for 30 min at 37°C, and 1:10, 1:100, and 1:1000 dilutions were plated onto Cassman blood agar, and incubated anaerobically at 37°C for 48 h. Adherence efficiency was calculated using the following formula:

$$\mathrm{Adherence efficiency}(\%)=\frac{\mathrm{Adherent bacterial cells}}{\mathrm{Initial inoculum}+\mathrm{adherent bacterial cells}}\times 100$$

### Statistical Analysis

The statistical significance (*p*-value) for all experiments was calculated with one-way ANOVA test. A Tukey *t*-test was used to analyze the different NTCD genotypes studied. A *p* < 0.05 was considered to be significant. Statistics were done with the ASTATSA calculators^1^.

## Results

### Isolation of NTCD Strains

A total of 74 *C. difficile* strains isolated from patients were tested for cytotoxicity and 14 of these strains were identified as NTCD strains, since they had no cytotoxic effect on either, Vero (Supplementary Figure S1) or HT29 cells (data not shown). Thus, in 19% of the clinical cases with hospital-acquired diarrhea we identified only infection with NTCD strains; of note, in these patients no mixed infection with toxigenic strains was found and no other enteropathogen was identified. Among these 14 strains 7 were isolated from adults and 7 from children. To corroborate the identity of these strains, all isolates were confirmed as *C. difficile* by PCR for the *16S rRNA* and *tpi* gene. Then the presence of toxin genes was analyzed by PCR, and we found the two previously reported genotypes (Natarajan et al., 2013): 9 strains with all three toxin genes absent (*cdtB^−^tcdA^−^tcdB^−^*) and 5 strains with all three genes present (*cdtB^+^tcdA^+^tcdB^+^)*. These results indicate that the lack of cytotoxicity is not always due to the absence of the genes. To further characterize these strains we also determined the presence of the *tcdC* gene in all strains and found that it was present in all 5 *cdtB^+^tcdA^+^tcdB^+^*, but absent in all 9 *cdtB^−^tcdA^−^tcdB^−^* strains (data not shown). Ribotyping of the 14 isolates showed that 4 of the 5 *cdtB^+^tcdA^+^tcdB^+^* strains were 027 type, whereas only one of the 9 *cdtB^−^tcdA^−^tcdB^−^* strains was 027 (Supplementary Figure S2). Clinical and genotype characteristics of the strains are present in Table 2.

**Table 2:** Clinical and genotypic characteristics of *C. difficile* non-toxigenic strains isolated from patients with hospital-acquired diarrhea.

		No. (%)	Strains
Sex	Female	7 (50.00)	MX019, MX025, MX066, MX107, MX113, MX125, MX154
	Male	7 (50.00)	MX041, MX151, MX153, MX184, MX249, MX367, MX501
Age	Adult	7 (50.00)	MX019, MX025, MX066, MX107, MX125, MX153, MX154
	Infant	7 (50.00)	MX041, MX113, MX151, MX184, MX249, MX367, MX501
Genotype	*cdtB^+^tcdA^+^tcdB*^+^/tcdC^+^	5 (35.71)	MX019, MX041, MX066, MX151, MX154
	*cdtB^−^tcdA^−^tcdB^−^/tcdC^−^*	9 (64.29)	MX025, MX107, MX113, MX125, MX153, MX184, MX249, MX367, MX501

### Colony Morphology

**FIGURE 1:**
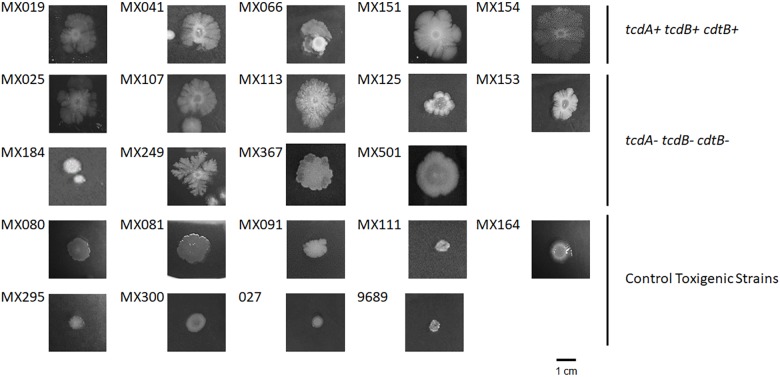
Colony morphology of non-toxigenic *C. difficile* (NTCD) strains. Strains were grown in BHI media for 7 days in anaerobically atmosphere as described previously. Bar = 1.0 cm.

Once we selected NTCD strains we compared their colony morphology after growing them for 5 days in BHI agar plates. Our results showed that colonies of NTCD were usually larger than colonies of toxigenic strains, and presented more ruffled edges (Figure 1), with some strains displaying highly branched colonies like strains MX113 and MX249. The size of NTCD colonies had an average of 1.89 cm, while toxigenic colonies had an average of 0.65 cm, a difference that was statistically significant (*p* < 0.05). Among the NTCD strains no difference in size was observed between those *cdtB^+^cdtA^+^tcdB^+^* and those *cdtB^−^cdtA^−^tcdB^−^*.

### Antibiotic Susceptibility

**FIGURE 2:**
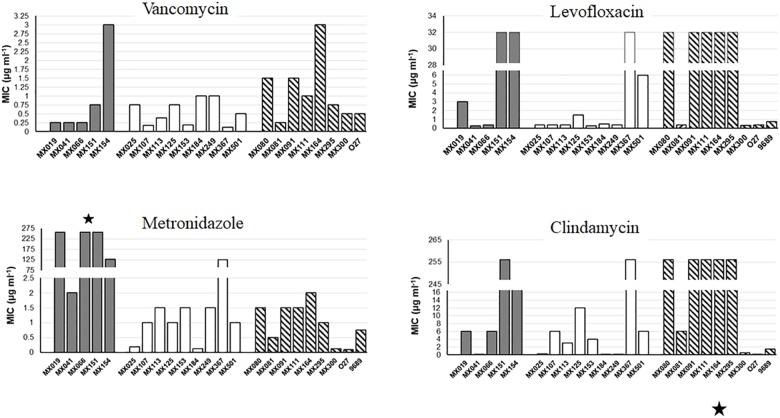
Antibiotic resistance of NTCD strains. E-Test analysis of NTCD strains. Gray bars: *cdtB*^+^*tcdA^+^tcdB^+^* strains. White bars: *cdtB^−^tcdA^−^tcdB^−^* strains. Striped bars: control toxigenic strains. ^∗^*p* < 0.05.

Non-toxigenic *C. difficile* strains were tested for sensitivity to clindamycin, metronidazole, levofloxacin and vancomycin using the threshold suggested by EUCAST and CLSI: >16 μg ml*^−^*^1^ for Clindamycin, >2.0 μg ml*^−^*^1^ for metronidazole, >2.0 μg ml*^−^*^1^ for vancomycin, and >4.0 μg ml*^−^*^1^ for levofloxacin. NTCD strains with the *cdtB^+^tcdA^+^tcdB*^+^ genotype showed a significantly increase resistance to metronidazole (*p* < 0.05) (Figure 2) when compared with *cdtB^−^cdtA^−^tcdB^−^* and with the cytotoxigenic strains. Cytotoxigenic strains presented higher resistance to levofloxacin and clindamycin than the NTCD strains, although difference did not reach statistical significance.

### Motility

**FIGURE 3:**
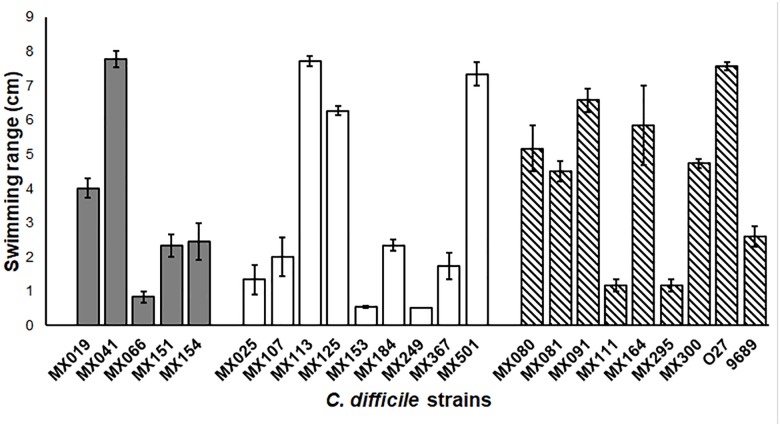
Non-toxigenic *C. difficile* motility. Range motility of *C. difficile* non-toxigenic and toxigenic strains. Cells were grown in soft BHI agar (0.15% agar) for 18 h, and swimming range was measure. Gray bars: *cdtB*^+^*tcdA^+^tcdB^+^* strains. White bars: *cdtB^−^tcdA^−^tcdB^−^* strains. Striped bars: control toxigenic strains. Values represent the average ± standard deviation (SD) from three independent experiments. *p* > 0.05.

In order to learn more about the phenotypic characteristic of NTCD strains, we analyzed the motility of the strains. The extent of the growth was measured after 18 h in BHI soft agar. Our results showed no difference in motility between the genotype and the capacity of swimming (Figure 3), although we found some strains that had an increased swimming ability (strains MX113, MX024, and MX041 and the 027 control strain), whereas others presented a very reduced motility (strains MX153 and MX249).

### Spore Formation in NTCD Strains

**FIGURE 4:**
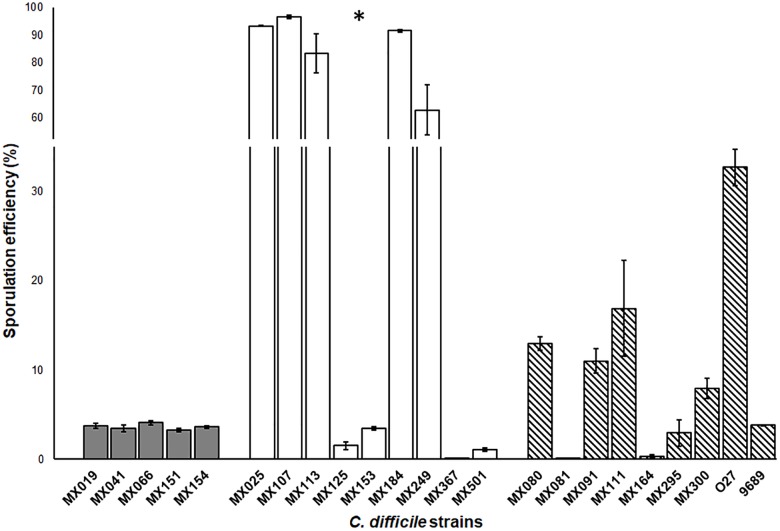
Sporulation efficiency of non-toxigenic *Clostridioides difficile* strains. Sporulation efficiency by chemical shock with 80% ethanol. Gray bars: *cdtB*^+^*tcdA^+^tcdB^+^* strains. White bars: *cdtB^−^tcdA^−^tcdB^−^* strains. Striped bars: control toxigenic strains. Values represent the average ± standard deviation (SD) from three independent experiments. ^∗^*p* < 0.05.

Sporulation is one of the most important advantages that *C. difficile* has as a pathogen. The spores provide the ability to resist disinfectants, antibiotics, hostile environment and are the vehicle for *C. difficile* transmission. Previous studies reported that sporulation levels of non-toxigenic strains were lower than the levels displayed by toxigenic strains (ref). In contrast, we found that whereas NTCD *tcdA^−^cdtB^−^tcdB^−^* strains showed a high capacity of spore formation, NTCD *tcdA^+^cdtB^+^tcdB*^+^ strains had a deficient spore formation capacity, and the toxigenic strains displayed a moderate capacity (*p* < 0.05) (Figure 4).

### Adherence of NTCD Strains in Vero Cells

**FIGURE 5:**
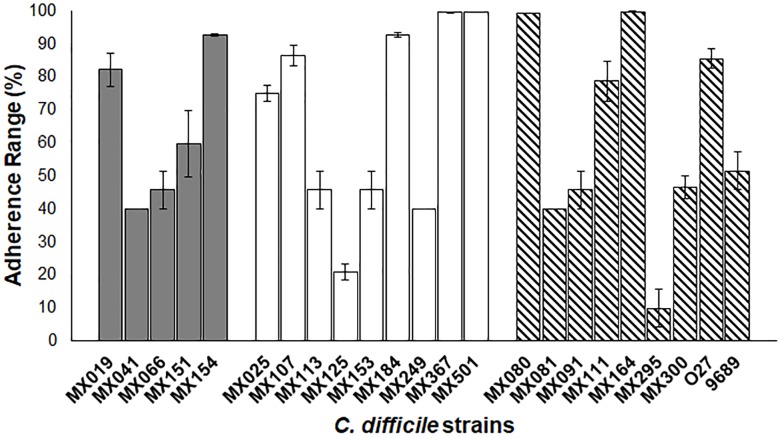
Adherence of NTCD strains in Vero cells. Monolayers of Vero cells were incubated with each strain. Cell adherence efficiency was measure as described previously. Gray bars: *cdtB*^+^*tcdA^+^tcdB^+^* strains. White bars: *cdtB^−^tcdA^−^tcdB^−^* strains. Striped bars: control toxigenic strains. The data presents and standard deviation are for three independent experiments. *p* > 0.05.

We analyzed the adherence ability of NTCD to Vero cells (Figure 5). The assay was done under anaerobic conditions as previously described. Results showed that most strains presented a high adherence efficiency, and only two had a reduced ability, below 20% (MX125 and MX295). There was no significative difference between genotypes and adherence.

## Discussion

Non-toxigenic *C. difficile* strains are poorly studied, and there is a need to learn more about the physiology and evolution of this type of *C. difficile* strains and to understand their role in disease. To our knowledge, this work is the first report on phenotypic characteristics of NTCD strains aiming to differentiate toxigenic from NTCD *C. difficile* strains. All the strains included in this work are clinical isolates from patients that presented hospital-acquired diarrhea and where no other enteropathogen was identified. In addition, in these cases we did not find mixed infection with toxigenic *C. difficile* strains, suggesting the possibility that NTCD strains may be associated with some cases of hospital-acquired diarrhea. This suggestion needs to be further studied by thoroughly characterizing the enterotoxicity of NTCD strains in cell and animal models.

In spite of the phenotypic heterogeneity of *C. difficile* strains, we were still able to identify some phenotypic characteristics that differentiates NTCD from toxigenic strains, in particular in their colony morphology, sporulation, and antimicrobial resistance. When we analyzed the colony morphology of NTCD strains, we identified that NTCD strains have more ruffled edges. Previous studies reported that this colony phenotype resulted after mutation in genes like the transcription–repair coupling factor, *mfd* (Willing et al., 2015b), the master regulator *spo0A* (Mackin et al., 2013), the transcriptional regulator *recV*, or the wall protein *cwpV* (Reynolds et al., 2011). Although it is known that Mdf protein is a repair factor, studies in *C. difficile* have shown that it may also have a role in transcriptional regulation, suppressing CodY and CcpA effect. In toxigenic strains, the mutation of *mdf* gene increase toxin production, and induce a change in colony morphology, although the factor responsible for this change in morphology is not known (Willing et al., 2015a). On the other hand, Spo0A protein has an important role in the control of sporulation (Underwood et al., 2009) and of toxin production in several strains (Mackin et al., 2013). A recent work reported that mutation of *spo0A* has an effect on colony morphology and motility across a solid surface, like the twitching motility mediated by type IV pili (Mackin et al., 2013). Thus, it is possible that NTCD strains may present mutations in genes that result in altered colony morphology but also in altered toxin production and in sporulation efficacy. In this sense, it is noteworthy that NTCD strains with the genotype *tcdA^−^cdtB^−^tcdB^−^* presented the highest efficacy in sporulation, although a previous study found no correlation between the genotype and spore variability (Burns et al., 2010). Sporulation may be regulated by as much as 314 genes (Fimlaid et al., 2013), and one of them is the *spo0A* gene. Our results in colony morphology and spore efficacy suggest that s*po0A* gene may be mutated in NTCD strains and that this mutation could also be responsible for the no-cytotoxic activity of these strains. The study of the genomes of NTCD strains will help clarify the genetic bases of these phenotypic characteristics; in this sense, we are in the process of sequencing the genomes of the NTCD strains reported in this work.

We observed that NTCD *tcdA^+^cdtB^+^tcdB*^+^ strains were more resistant to metronidazole than the *tcdA^−^cdtB^−^tcdB^−^* and the toxigenic strains. The resistance of *C. difficile* toxigenic strains to metronidazole has been previously reported, and it has been suggested that this may have a high impact on the pathophysiology of recurrent CDI diseases (Richardson et al., 2015). There is a report on a single NTCD strain with a stable resistance to metronidazole where resistance was associated with an altered *pfo* gene expression. Pfo is a pyruvate-flavodoxin oxidoreductase that participate in the response to oxidative stress (Moura et al., 2014). Since in our study most metronidazole resistant strains were NTCD *tcdA^+^cdtB^+^tcdB*^+^, it will be interesting to see if there is any association between resistance to metronidazole and inhibition of toxin expression.

The ability to adhere to cells and the motility were phenotypes that were not different among the strains that we studied, regardless of cytotoxicity or genotype. Previous studies have shown that some toxigenic strains that do not have flagella, they still conserve late flagellar genes that are also responsible for regulation of toxin production (36). It will be of interest to study the role of flagellar genes in our NTCD strains to better understand the processes of cytotoxicity, motility, adherence, and colonization in *C. difficile*.

The natural history of the infection by C. difficile NTCD strains remains unclear, and their participation in the pathogenesis of intestinal disease is yet unknown. In this study we characterized NTCD strains isolated from patients with hospital-acquired diarrhea, which suggest that they could cause disease even in the absence of toxin production. We identified two NTCD genotypes, one PCR positive for all three toxins (tcdA^+^cdtB^+^tcdB^+^) and the other PCR negative for the toxins (tcdA^−^cdtB^−^tcdB^−^). We do not know if tcdA^+^cdtB^+^tcdB^+^ strains have functional genes and are able to produce toxin in vivo in the human intestine. Of note, we detected the presence of the tcdC negative regulator gene in the 5 tcdA^+^cdtB^+^tcdB^+^ NTCD strains, which suggest that probably these strains have a deficient positive regulator cdtR gene. On the other hand, the strains tcdA^−^cdtB^−^tcdB^−^, may be able to produce unknown virulence factors, which elucidation requires further studies. Also, we found that most of the tcdA^+^cdtB^+^tcdB^+^ NTCD strains were of the ribotype 027, the ribotype most commonly reported for toxigenic strains. Our results suggest 027 is common in strains with the toxin genes, even if the show negative for cytotoxicity. On the other hand, most of the tcdA^−^cdtB^−^tcdB^−^ were not 027, which is also in agreement with previous reports (Urban et al., 2001; Pereira et al., 2016; Riley et al., 2018). Only one isolate (strain 153) was negative for toxins by PCR but still ribotype 027, a result that is not agreement with previous reports (Valiente et al., 2014); and we need to further study this isolate. A work in progress is the sequencing of the genome of these NTCD isolates, which will help clarify if this strain has truncated or mutated toxin genes that make it PCR negative.

## Conclusion

In spite of the diversity in the phenotypic characteristics among the studied strains, there were significant differences in colony morphology, metronidazole resistance, and sporulation between NTCD and cytotoxigenic strains. Colony morphology and sporulation differentiated between NTCD and cytotoxigenic strains, whereas metronidazole resistance differentiated NTCD *tcdA^+^cdtB^+^tcdB*^+^ strains from NTCD *tcdA^−^cdtB^−^tcdB^−^* and from toxigenic strains. We are currently sequencing the genome of both toxigenic and NTCD strains to better understand the genomic characteristic of both types of strains.

## Ethics Statement

This project was evaluated and authorized by the Bioethics Committee of the “Hospital de Pediatría, Instituto Mexicano del Seguro Social” with the registration number R2015-785-089. All participants provided written informed consent, and in the case of children under 16 years, their parents were responsible for it.

## Author Contributions

MR-C and JT designed the experiments. MR-C and MS-R carried out all experiments. MR-C, MC, and JT wrote the manuscript and analyzed the data. All authors have critically read, corrected, and approved the final version of the manuscript.

## Conflict of Interest Statement

The authors declare that the research was conducted in the absence of any commercial or financial relationships that could be construed as a potential conflict of interest.

## Footnotes

1 http://astatsa.com/OneWay_Anova_with_TukeyHSD

## Supplementary Material

FIGURE S1 Cytotoxic analysis of *C. difficile* strains in Vero cells. After cytotoxic analysis, cells were washed with PBS and fixed with methanol. Then Giemsa staining was performed to analyze the cytotoxic effect in Vero cells.

FIGURE S2 Ribotyping of NTCD strains. PCR ribotype analysis from the non-toxigenic strains from this study.
