# Supplementary figures and images for: Phenotypic Characterization of Non-toxigenic Clostridioides difficile Strains Isolated From Patients in Mexico
Source: Front Microbiol. 2019 Feb 1;10:84. doi: 10.3389/fmicb.2019.00084 (PMC6367242; doi:10.3389/fmicb.2019.00084)

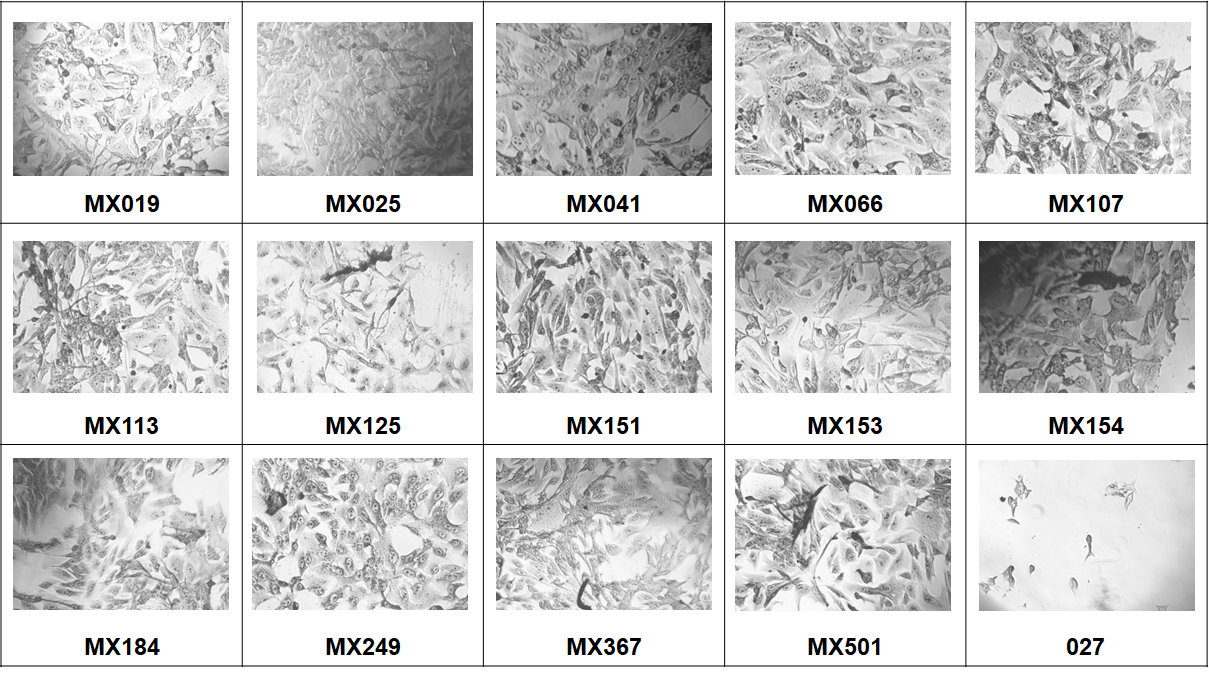

Supplement: FIGURE S1 — Cytotoxic analysis of C. difficile strains in Vero cells. After cytotoxic analysis, cells were washed with PBS and fixed with methanol. Then Giemsa staining was performed to analyze the cytotoxic effect in Vero cells. [file Image_1.TIF]

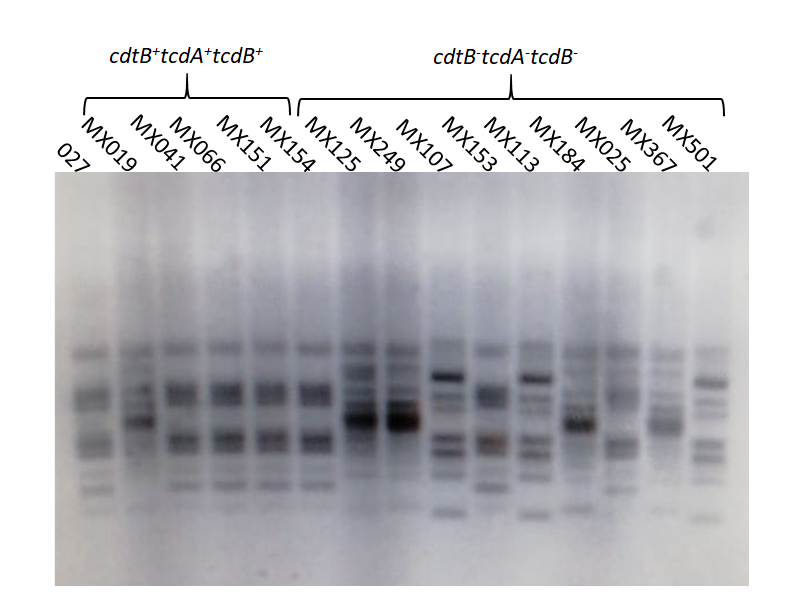

Supplement: FIGURE S2 — Ribotyping of NTCD strains. PCR ribotype analysis from the non-toxigenic strains from this study. [file Image_2.TIF]
